# DNA Mismatch Repair Gene Variants in Sporadic Solid Cancers

**DOI:** 10.3390/ijms21155561

**Published:** 2020-08-03

**Authors:** Fabian Caja, Ludmila Vodickova, Jan Kral, Veronika Vymetalkova, Alessio Naccarati, Pavel Vodicka

**Affiliations:** 1 Department of Immunotherapy, Institute of Microbiology of the Czech Academy of Sciences, 14200 Prague, Czech Republic; caja.fabian@gmail.com; 2 Department of Cell Biology, Faculty of Science, Charles University, 12000 Prague, Czech Republic; 3 Department of Molecular Biology of Cancer, Institute of Experimental Medicine of the Czech Academy of Sciences, 14220 Prague, Czech Republic; ludmila.vodickova@iem.cas.cz (L.V.); krlj@ikem.cz (J.K.); veronika.vymetalkova@iem.cas.cz (V.V.); alessio.naccarati@hugef-torino.org (A.N.); 4 Institute of Biology and Medical Genetics, 1st Medical Faculty, Charles University, 12000 Prague, Czech Republic; 5 Biomedical Centre, Faculty of Medicine in Pilsen, Charles University in Prague, 32300 Pilsen, Czech Republic; 6 Department of Gastroenterology and Hepatology, Institute for Clinical and Experimental Medicine, 14200 Prague, Czech Republic; 7 Italian Institute for Genomic Medicine (IIGM), 10060 Torino, Italy

**Keywords:** mismatch repair, genetic variants, genes, genotype, single nucleotide polymorphism, cancer, patients, treatment outcome

## Abstract

The phenotypic effects of single nucleotide polymorphisms (SNPs) in the development of sporadic solid cancers are still scarce. The aim of this review was to summarise and analyse published data on the associations between SNPs in mismatch repair genes and various cancers. The mismatch repair system plays a unique role in the control of the genetic integrity and it is often inactivated (germline and somatic mutations and hypermethylation) in cancer patients. Here, we focused on germline variants in mismatch repair genes and found the outcomes rather controversial: some SNPs are sometimes ascribed as protective, while other studies reported their pathological effects. Regarding the complexity of cancer as one disease, we attempted to ascertain if particular polymorphisms exert the effect in the same direction in the development and treatment of different malignancies, although it is still not straightforward to conclude whether polymorphisms always play a clear positive role or a negative one. Most recent and robust genome-wide studies suggest that risk of cancer is modulated by variants in mismatch repair genes, for example in colorectal cancer. Our study shows that rs1800734 in *MLH1* or rs2303428 in *MSH2* may influence the development of different malignancies. The lack of functional studies on many DNA mismatch repair SNPs as well as their interactions are not explored yet. Notably, the concerted action of more variants in one individual may be protective or harmful. Further, complex interactions of DNA mismatch repair variations with both the environment and microenvironment in the cancer pathogenesis will deserve further attention.

## 1. Introduction

### 1.1. DNA Mismatch Repair System and Its Role in Tumorigenesis

The DNA mismatch repair system (MMR) is an integral part of the DNA damage response pathway (DDR), responsible for maintenance of genomic integrity. MMR preferably corrects frameshift mutations in microsatellites and mismatched nucleotides generated during DNA replication [1,2]. The importance of MMR in replication fidelity is illustrated in Table 1. Depending on the type of DNA damage, MMR also interacts with DDR and therefore triggers cell cycle arrest and apoptosis [3]. Impaired MMR function results consequently in DNA damage accumulation and mutational load, leading to DDR disruption and genomic instability. It has been postulated that every DDR process is functionally affected during carcinogenesis [4]. Defects in DDR genes are particularly apparent in hereditary and familial cancers with known high penetrance germline mutations in DNA repair genes [5,6,7,8,9]. For instance, loss of function of MMR (the so-called mutator phenotype) due to germline mutations is responsible for hereditary non-polyposis colorectal cancer (also Lynch) syndrome [6,10]. Genetic changes in the MMR system underlie predisposition and etiopathogenesis to cancers of the colon, endometrium, ovary and other organs [8,9,11], recently reviewed by Valle et al. [12]. While rather rare germline mutations in MMR (*MSH2/MLH1*) genes exert high effect in the above-mentioned hereditary syndromes, sporadic malignancies arise as an interplay of multiple common alleles (including those in MMR system) with rather modest effect [2,13]. As the overwhelming majority of malignancies are of sporadic (non-familial) origin, these common variants in individual’s genetic material that predispose to higher cancer risk and its further progression are currently of importance. Variations in MMR genes are common in the general population and may be associated with a moderate increase in cancer risk [14]. Both coding and non-coding MMR genetic variants may disrupt the physiological function of MMR proteins; even alterations that do not cause a change in amino acid sequence could lead to aberrant splicing, changes in transcription regulation, modifying microRNA binding, etc. [15]. All these variants are in important interplay with environmental, microenvironmental and lifestyle factors. Especially the combination of SNPs considered as less pathogenic in one individual may increase the risk of cancer in the others [16,17,18]. Finally, genetic variants may also modulate the response to therapy and drug toxicity [19].

### 1.2. Function of DNA Mismatch Repair System

The S-phase of the cell cycle is characterised by DNA replication and the duplication of the whole genome. The replicative polymerases may incorporate inappropriate nucleotides leading to base–base mismatches or they can slip on nucleotide sequences, creating insertion–deletion loops (IDLs). This erroneous activity of DNA polymerases generates single nucleotide point mutations or frameshift mutations resulting in the synthesis of truncated protein. Arising biosynthetic errors in newly synthesised DNA, not corrected by exonucleases, are removed by the MMR. In prokaryotes, it involves three specialised proteins: MutS, MutL and MutH. MutS is an ATPase acting as a homodimer or heterotetramer, which recognises base–base mismatches and small IDLs [21,22,23]. Its loading onto the DNA leads to conformational change allowing its interaction with MutL, an ATP-dependent heterodimer essential for removal of DNA fragment containing the mismatch [24]. Finally, MutL interacts with another MMR component MutH and activates its endonuclease activity. It nicks unmethylated strain of hemimethylated DNA. MutH has no homolog in eukaryotes, its 5′→3′endonuclease activity is taken up by MutL homologs [25,26].

**Figure 1:**
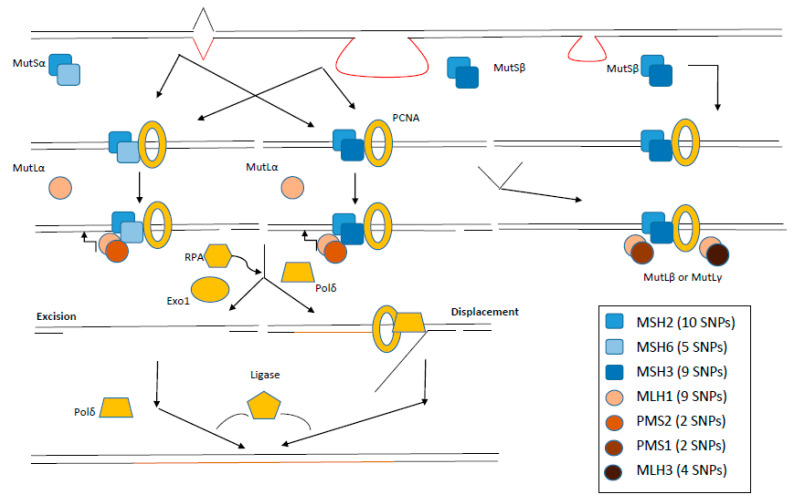
Molecular principles of the eukaryotic MMR system. MutSα protein complexes recognise mainly nucleotide mismatches, whereas MutSβ complexes recognise insertion–deletion loops. Further steps of MMR include interaction with MutL protein complexes and other proteins (exonucleases, replication proteins and polymerases). Numbers in brackets summarise the number of known pathogenic SNPs with F ≥ 5% in EUR population (adapted from the work in [34,35]). PCNA: Proliferating cell nuclear antigen; EXO1: Exonuclease 1; Pol δ: DNA polymerase δ; RPA: Replication protein A.

The eukaryotic MMR system (Figure 1) consists of following homologs; five MutS (MSH2–MSH6), two MutL (MLH1, MLH3) and three postmeiotic-segregated proteins (PMS1, PMS2 and PMS2L). MSH2 forms a heterodimer with MSH6 or MSH3 giving rise to the assembly of MutSα and MutSβ complexes, respectively [27,28]. MutSβ complexes repair preferentially heteroduplexes with larger IDLs than MutSα complexes [29,30]. In humans, MLH1 is the major MutL homolog which assembles to MutLα, MutLβ and MutLγ complexes with PMS2, PMS1 and MLH3 proteins, respectively [31,32]. SNPs were identified in practically all MMR genes, predominantly in *MLH1* and *MSH2* (Figure 1).

In fact, the MMR system is so vital for the integrity of the human genome that low penetrance variations are strongly selected. It is very unlikely that these alleles can drift to polymorphic level. However, a combination of more polymorphisms with minute effect may disrupt cellular responses to DNA damage and alter individual’s sensitivity to carcinogens [16,33]. They may modulate tumour occurrence, growth, metastatic progression and response to chemotherapy. However, there is still a gap in understanding the real function of polymorphisms in the human genome and their impact on the cancer development. Here, we overview the possible role of SNPs in MMR genes in susceptibility to and development of cancer, as well as in the prognosis and treatment prediction.

## 2. Materials and Methods

**Figure 2:**
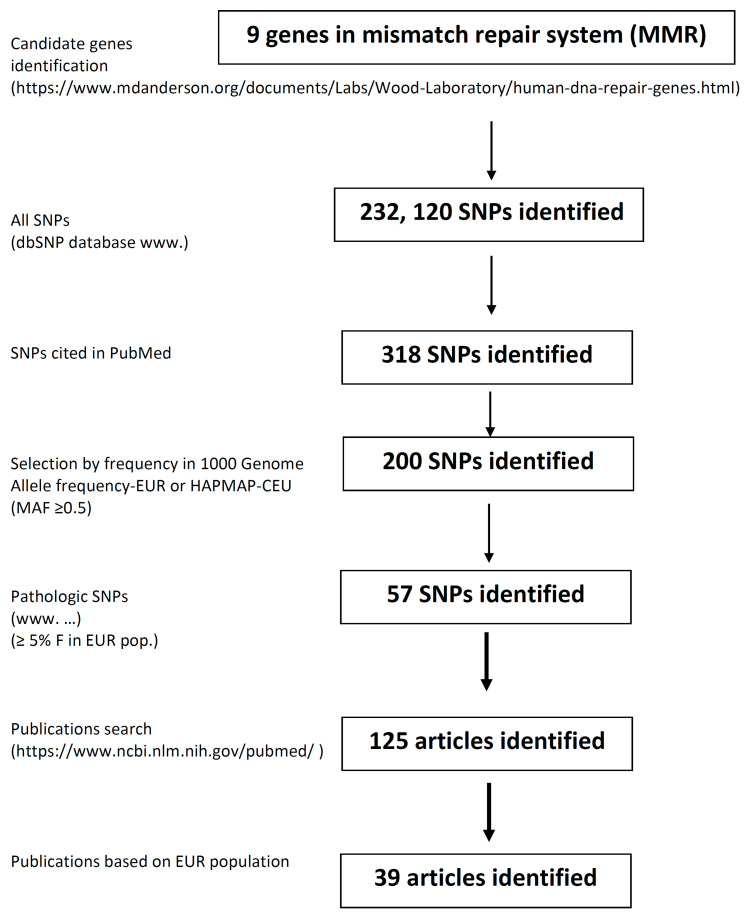
Workflow of the study.

The PubMed database was searched for the results of all studies published before July 2020, using the keywords “mismatch repair gene”, “polymorphisms”, “genetic variant”, “sporadic cancer”, “single nucleotide polymorphism” and “genome-wide association studies”. Relevant papers were selected and critically analysed. The dbSNP database (http://www.ncbi.nlm.nih.gov/snp/) was used as the primary source of all SNPs. All human MMR genes were also analysed for SNPs in open access SNP databases, when additional information was needed: F-SNP database of Queen’s University (http://compbio.cs.queensu.ca/F-SNP/), Canadian MutDB of The Buck Institute Mooney Lab (http://mutdb.org/) and in British In-SIGHT (The International Society for Gastrointestinal Hereditary Tumours Incorporated) database (http://www.insight-group.org/). Relevant papers cited in these databases were also assessed and used in the review. We have concentrated on papers describing the role of SNPs in human MMR genes in the development of primary sporadic solid cancers. We prepared our own database that included those selected SNPs with their frequency in all populations worldwide. In this review, however, we focused on the European population primarily and considered the minimal population frequency of SNP exceeding 5%. We also considered the pathogenicity of SNPs, which were studied in non-European populations only, but their frequency in the European population is equal to or more than 5%. All other SNPs, even pathogenic in other populations, but with lower allele frequency in the European population, were not included in this review. Additional information about SNP reference numbers, gene location, amino acid change, phenotypic change prediction, minor allele frequency in the European and other populations and references to literature is available in the Supplementary Materials. All SNPs, either with an adverse or protective effect on the onset of cancer, were summarised by Venn diagrams. SNPs that had the same impact for various types of cancer were localised in the centre of the diagram, and SNPs with more specific impact were localised more peripherally. This review focuses primarily on genes encoding parts of MMR protein complexes MutL and MutS; other MMR-related proteins, such as Proliferating cell nuclear antigen (PCNA), Exonuclease 1 (EXO1), DNA polymerase δ (Pol δ) and Replication protein A (RPA), have not been considered. The workflow of this study is presented in Figure 2. The functionality of SNPs was ascertained by using in silico approaches. The phenotypic effect of the SNP on the functionality of the protein was inferred on the basis of software, such as SIFT, PolyPhen and PupaSuite.

## 3. Results on MMR Gene Variants

### 3.1. MLH1 Gene (Mutl Homolog 1)

The *MLH1* gene, located on chromosome 3 at the p22.2 site, encodes substantial protein of the MMR system. Twenty-three transcripts have been identified so far. The longest NM_000249 is coding for a 756 amino acid protein MLH1, which forms heterodimers with other MMR proteins, including PMS2, PMS1 and MLH3. *MLH1* is the most frequently mutated MMR gene, in both sporadic and hereditary cancers. *MLH1* gene mutations represent the most prevalent cause of an inherited form of colorectal cancer (CRC), hereditary non-polyposis colorectal cancer (HNPCC) [12,36]. According to the comprehensive dsSNP server, the human *MLH1* gene contains 15,721 SNPs. However, only 49 SNPs were cited in the PubMed database. 

Apart from coding SNPs and variants in regulatory regions for corresponding gene expression, there are MMR variants that may also interact with epigenetic silencing (e.g., methylation), contributing further to gene expression regulation with implications on the MMR system [37].

**Figure 3:**
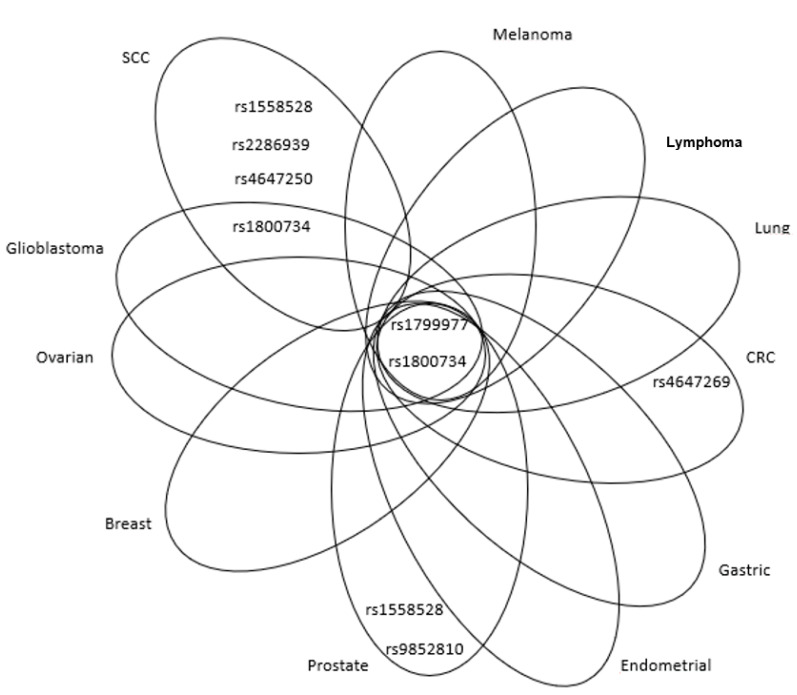
Venn diagram showing relevant MLH1 SNPs, associated with adverse effects, in overlap among different cancer types or peculiar for specific malignancies. SCC: squamous cell carcinoma; CRC: colorectal cancer.

SNPs located in the promoter region of the *MLH1* gene are sufficient to affect the expression of the gene. For example, the most intensively studied intron SNP rs1800734 (c.-93G>A, upstream variant near 5′ untranslated region) was repeatedly described as pathogenic, increasing the risk of various types of cancer, sporadic CRC and endometrial cancer in particular [38,39,40,41,42,43]. The SNP is located in the region essential for the maximum transcriptional activity. For instance, the A/A genotype was associated with the increased risk of glioblastoma (odds ratio (OR) = 3.14, 95% confidence interval (CI) = 1.09–9.06, *p* = 0.034) [44]. This SNP also increases the risk of lung adenocarcinoma in European, Korean and Taiwanese populations [45,46,47,48]. One European study described the association between the A/A genotype of this SNP and lung cancer in interaction with chromium exposure [48]. The accumulated evidence on the pathogenicity of this SNP in the lung cancer is highlighted in a meta-analysis comprising 1018 publications and 754 genes [49]. The precise mechanism by which rs1800734 downregulates the function of MLH1 is unknown, but some mechanisms have been suggested. The variant is localised in the promoter region in the consensus sequence binding transcription factor AP-4, which is important for the initiation of transcription [47]. Raptis et al. found this variant in two patients with the microsatellite instability-high (MSI-H) form of CRC. The rs1800734 variant was also associated with increased promoter methylation, suggesting the existence of the sequence specificity for promoter methylation [50]. Indeed, the risk allele of the SNP rs1800734 showed a strong association with *MLH1* promoter hypermethylation and loss of MLH1 protein in CRC tumours [37]. Recently, Ning Qing Liu et al. reported the A-allele of rs1800734 within the promoter region of *MLH1* as perturbing the binding of allele-specific transcription factor AP4 and consequently increasing *DCLK3* expression through a long-range interaction, which promotes malignant transformation through enhancing expression of the genes related to epithelial-to-mesenchymal transition [51]. An additional study confirmed that rs1800734 is a functional polymorphism that results in decreased *MLH1* transcription [52]. However, it has to be considered that different effects of SNPs on the function of the gene may emerge in different human populations. On the other hand, Pan et al. proved in a meta-analysis comprising the data from 17 studies that SNP rs1800734 was not associated with an increased risk of cancer [53]. The authors noted the following peculiarity. Low-quality studies were associated with a higher risk than high-quality studies and hospital-based studies showed a higher risk than population-based studies. Interestingly, a more pronounced risk was observed in the Asian population than in the Caucasian population. The divergent results were provided by the studies on CRC patients. While rs1800734 increased the risk of CRC, the same SNP decreased the risk of rectal cancer alone [54]. The rs1800734 polymorphism within the core promoter region of *MLH1* gene has been reported to increase the risk of microsatellite instable (MSI-H) CRC (OR = 1.39; *p* = 1.45 × 10^−4^), as assayed for in 3132 cases. A meta-analysis further strengthened the above evidence for rs1800734 as an event in a specific CRC subtype (*p* = 3.43 × 10^−12^) [40]. Finally, a recent study based on genome-wide association studies (GWAS) data by Pardini et al. comprising over 27,000 individuals showed rs1800734 to be significantly associated with the risk of colon cancer (OR = 1.13, 95% CI = 1.07–1.18, *p* = 3.48 × 10^−6^) [55]. When looking at tumour site, rs1800734 was mainly associated with proximal colon tumours (OR = 1.13, 95% CI = 1.07–1.18). Due to many differences in embryogenesis, etiology, anatomy, genetics, and treatment response between the colon and rectal tumours, CRC is no longer a single etiopathogenic entity. The studies reporting the association of rs1800734 with cancer are provided in the Supplementary Materials. The summary of the adverse effects of SNPs in *MLH1* is shown in Figure 3. Our recent review pointed out that promoter *hMLH1* hypermethylation is associated with CIMP (CpG island methylation phenotype) and acts as main mechanism for the evolution of sporadic MSI CRC [56]. Interestingly, the rs1800734 polymorphism within the core promoter region of *MLH1* gene has been reported to increase a risk of microsatellite instable (MSI-H) CRC, accounting for ~20% of cases. Variation in the 5′-UTR region of *MLH1* (Table 2) is likely to be associated with methylation status of this gene (variant allele linked with hypermethylation) and increase the risk of various malignancies (Figure 3). Whether this effect is mediated sterically by variation or via the altered activity of DNA methyltransferase remains to be elucidated.

Additionally, analysis of a region of chromosome 3p21 spanning the MLH1 locus in peripheral blood cells of healthy individuals showed that a CpG island shore 1 kb upstream of the *MLH1* gene evinces differential methylation profile following the stratification by *MLH1* variants (rs1800734, rs749072 and rs13098279). Significantly higher white blood cell shore methylation has been reported in individuals with wild type genotypes as compared to those with heterozygous or homozygous genotypes [57]. Although the differences in *MLH1* promoter methylation may not solely explain the increased risk of several cancers exerted by rs1800734, this epigenetic mechanism should not be overlooked.

Another intensively studied SNP in the *MLH1* gene, rs1799977 (c.655A>G; Ile219Met), is located in exon 8 [58]. This SNP was often ascribed as harmful and associated with increased risk of various types of cancer (summarised in the Supplementary Materials). Nejda et al. found that rs1799977 elevates the risk of sporadic CRC but it also provides better outcomes for CRC patients: G allele carriers displayed a lack of vascular invasion, less frequent distant metastases and reduced recurrence than wild type A carriers [59]. Taken together, rs1799977 is considered as a SNP associated with the increased risk of CRC, breast (BC) and lung cancer; invasive, but not serous, ovarian cancer; prostate cancer; and also with increased risk of death from B cell lymphoma [41,60,61,62,63,64]. On the contrary, some authors found that homozygous carriers of rs1799977 also had lower risk of BC and lung cancer in a Caucasian population [60,65]. Although SNP rs1799977 has no significant impact on the MLH1 function in vitro, it is associated with many functional defects in vivo. For instance, Campbell et al. proposed that it is statistically significantly associated with the Western diet and smoking (*p* = 0.03 and *p* = 0.005, respectively) [66]. Patients with Ile/Ile amino acid change, who consumed more red and processed meat, eggs, high-fat foods, refined grains and added sugars, conferred a higher risk of CRC. In summary, it is evident that SNP rs1799977 should be considered individually in each population for its impact on cancer risk. For example, the investigation of rs1799977 in almost 1000 patients of Czech origin, which became a part of an extensive international meta-analysis, showed no association with the risk of sporadic CRC [67]. However, this SNP should be analysed separately in patients with colon and rectal cancer as discussed earlier. It was observed that rs1799977 carriers have a higher risk of CRC and display the expression imbalance of *MLH1*, but the same SNP has a protective effect in rectal cancer patients [41,59,68]. 

Interesting results were obtained from the multi-gene studies. The role of SNPs in various genes associated with the CRC risk was intensively studied in the Czech population due to the high incidence of the disease in this area. The study of Tulupova et al. included five MMR genes which were genotyped in 614 cases and 614 matched controls for 10 SNPs. For *MLH1*, the authors focused on an intronic variant, rs4647269 (c.791-1406C>T), and observed that its carriers exhibited a decreased risk of rectal cancer (OR = 0.71, 95% CI = 0.51–0.9, *p* = 0.04) [54]. The same study found an intronic SNP of *MSH6i*, rs3136228 (c.-152-405T>G), to be associated with increased risk of CRC (OR = 1.29, 95% CI = 1.02–1.62) and exonic rs1042821 (c.116G>C, Gly39Ala) with decreased risk of CRC (OR = 0.76, 95% CI = 0.60–0.98). Rs2072447 (c.3438+14A>T) was associated with increased risk of colon cancer (OR = 1.34, 95% CI = 1.03–1.74). Authors observed a differential distribution of haplotypes based on three *MSH6* SNPs in the cases and controls (global *p* = 0.02). The TAG haplotype was associated with a decreased risk of CRC (OR = 0.74, 95% CI = 0.59–0.92), whereas the most frequent haplotype GGG with increased risk of rectal cancer (OR = 1.32, 95% CI = 1.05–1.65) [54]. Another population-based study analysed the association of SNPs in MMR genes with prostate cancer risk [62]. A population (1484 cases) included Caucasian and African-American men, who were diagnosed with prostate cancer between 1993 and 2005. Among Caucasians, the authors disclosed a strong association between rs9852810 (c.1410-1306G>A) and overall prostate cancer risk (OR = 1.21, 95% CI = 1.02–1.44, *p* = 0.03), more aggressive prostate cancer (OR = 1.49, 95% CI = 1.15–1.91, *p* < 0.01) and prostate cancer recurrence (hazard ratio (HR) = 1.83, 95% CI = 1.18–2.86, *p* < 0.01). The authors also reported more aggressive prostate cancer in association with rs1799977 [62]. This SNP has not been analysed for its pathogenicity in prostate cancer in other populations so far. A marginal association between a risk of squamous cell type lung cancer and intronic variants rs4647250 (c.453+544T>C), rs1558528 (c.67+955C>A) and rs2286939 (c.1038+86T>C) was observed in a Chinese population, *p* = 0.04 for all these SNPs [61]. The same study also found an association between the G allele of rs1799977 and the increased risk of lung cancer (OR = 3.65, 95% CI = 1.44–9.24, *p* = 0.006); particularly among younger patients (OR = 5.28, 95% CI = 1.45–19.21, *p* = 0.01). Only few SNPs had the protective effect in various cancers, for example, rs2286940 (c.1410-169C>T) was associated with the better overall survival (OS) of lymphoid cancer patients (HR = 2.06, 95% CI = 1.35–3.16, *p* < 0.001) and rs1540354 (c.307-1403A>T) with the better OS of CRC patients (HR = 1.90, 95% CI = 1.14–3.17, *p* = 0.01) [69,70]. A GWAS study on 2795 predominantly Caucasian women and 4505 controls addressed SNP–SNP interactions (based on a priori knowledge for potential epistatic interactions) in BC susceptibility. The authors observed two-way SNP–SNP interactions (*APEX1*-rs1130409 and *RPAP1*-rs2297381; *MLH1*-rs1799977 and *MDM2*-rs769412) that conferred elevated risks for BC (*p* (interaction) < 7.3 × 10^−3^) [71]. Figure 3 illustrates that SNPs rs1799977 and rs1800734 in *MLH1* exert adverse effects on the risk of several cancer, except for squamous cell carcinoma (SCC). No consistent tendency may be tracked regarding the protective effects of *MLH1* polymorphisms. 

### 3.2. MLH3 Gene (Mutl Homolog 3)

**Figure 4:**
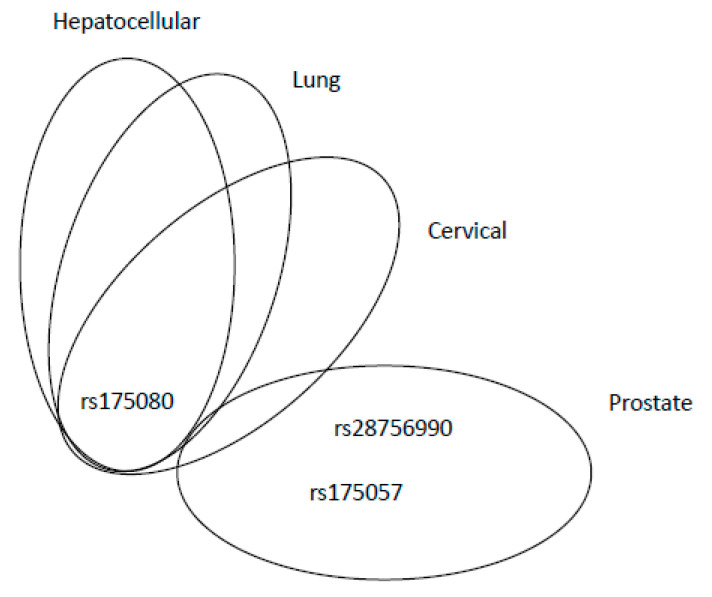
Venn diagram showing relevant MLH3 SNPs, associated with adverse effects, in overlap among different cancer types or peculiar for specific malignancies.

The *MLH3* gene is located on chromosome 14 at q24.3 site. Thirteen transcripts of this gene have been described so far. The longest one is coding a 1453 amino acid protein MLH3, which forms a heterodimer with MLH1 (MutLγ complex), involved in meiotic crossing-over [72]. The other two MutL complexes, MutLα and MutLβ, do not consist of MLH3. According to the dbSNP server, 9007 SNPs were identified in human *MLH3*, but only 15 SNPs were cited in PubMed database. The majority of *MLH3* gene SNPs is localised in extraordinarily long exon 2, which contains 3343 base pairs (bps). SNP rs175080 (c.2531C>T, Leu844Pro) was studied more exclusively. Conde et al. found that either the homozygous or heterozygous variant was associated with decreased risk of BC [73]. Authors also recorded that the combination of rs175080 with a SNP in *MSH4* gene rs5745325 (c.289G>A, Ala97Thr) is pathogenic, being associated with an increased BC risk. MSH4 is a meiosis-specific protein, and it is probable that MLH3–MSH4 interaction is crucial for meiotic recombination [74]. The abrogated interaction between these two MMR proteins may influence the recombination process, resulting in an increased recombination rate in mammary gland cells. Interestingly, another study described the rs175080 SNP as a variant increasing the risk of lung cancer, but in interaction with other risk factors, such as smoking [75]. The association between the AA genotype of rs175080 and a higher risk of the primary hepatocellular carcinoma was observed in a Chinese population [76]. This SNP increased the risk of cervical carcinoma, as assayed for on a Chinese population as well [77]. Comprehensive pathway-based analysis of the combined dataset of two pancreatic cancer GWAS, PanScan 1 and PanScan 2, found an intronic SNP rs175057 (c.4012-37G>A) to be associated with the increased risk (*p* = 0.039) of pancreatic cancer in a US population [78]. A GWAS performed on a Chinese population found an association of rs28756990 (c.2221G>A, Val741Ile) with prostate cancer risk [79]. All other known SNPs in *MLH3* were not studied for their role in the carcinogenesis. The overview of adverse effects of *MLH3* SNPs for individual malignancies is shown in Figure 4: rs175080 has an adverse effect on hepatocellular, lung and cervical cancers, whereas rs28756990 and rs175057 were associated with prostate cancer only. Interestingly, rs175080 had a protective role in BC.

### 3.3. MSH2 Gene (MutS Homolog 2)

The *MSH2* gene is located on chromosome 2, at the p21-p16.3 site. There are six transcripts of the gene; the longest one with 16 exons encodes a protein MSH2 encompassing 934 amino acids. The dsSNP database comprises 68,322 SNPs in *MSH2*, 83 of them cited in PubMed. Functionally, MSH2 is one of the most important protein components of post-replicative DNA mismatch repair system. Together with MSH6 and MSH3 it forms MutSα and MutSβ complexes, respectively. Briefly, MutSα repairs single-base mismatches or dinucleotide insertion-deletion loops, whereas MutSβ restores of long insertion–deletion loops. MutS complexes associate with MutLα heterodimers, involved in downstream repair events. MutSα also participates in the DNA homologous recombination repair and it regulates in melanocytes cell cycle regulation and apoptosis [80,81].

No exonic SNP in *MSH2* was associated with the cancer risk (see Supplementary Materials). A recent study revealed the association between SNP rs2303425 (c.-68-50T>C), localised in 5′ UTR region, and increased risk of lung cancer (OR = 2.28, 95% CI = 1.12–4.63, *p* = 0.024) in a Slovak population [82]. The same study showed on 422 cases and 486 controls that rs1800734 in *MLH1* was associated with increased lung cancer risk (OR = 1.40, 95% CI = 1.08–1.82, *p* = 0.01); the interaction between rs1800734 and rs2303425 (*MSH2*) revealed elevated risk for genotype GG/CC (OR = 3.08, 95% CI = 1.09–8.72, *p* = 0.03), which was further pronounced in females (OR = 11.56, 95% CI = 1.33–100.36, *p* = 0.005). The SNP rs2303425 in *MSH2* is also associated with the higher risk of the luminal A subtype of BC in a Taiwanese population [83]. Individuals bearing C/C genotype had a significantly higher risk of BC compared to those with the T/T genotype (OR = 2.0; 95% CI = 1.1–3.8, *p* < 0.05). The functional effect of this SNP on *MSH2* expression was studied by luciferase assay, which showed that the cells with C/C genotype had significantly reduced expression of the *MSH2* gene. The authors discussed that this SNP is located in NF-Y transcription factor binding site, also known as an estrogen-responsive element and might reduce the binding of the estrogen to the promoter of *MSH2*, resulting in its decreased activity [83]. 

Tumours of the gastrointestinal tract (GIT) are associated with the SNP rs1981929 (c.1277-118G>A), where variant allele confers increased risk of CRC; rs2303426 (c.211+9C>G) was associated with the gallbladder, oral and head and neck cancers, and rs10183143 (c.1661+90T>C) posed a risk for gastric cancer [84,85,86,87,88]. The gastric cancer risk was further increased by drinking alcohol and eating the pickled and fried food [88].

An intronic SNP rs2059520 (c.2006-265A>G) was found to be associated with endometrial cancer risk (*p* = 0.03) [39]. Increased risk of this type of cancer was also observed for SNP rs2303428 (c.2006-6T>A). The authors showed that women with binary SNP interaction of rs2059520 or rs2303428 with rs1800734 in *MLH1* (accounting for ~9% of all endometrial cancer cases) had a particularly higher risk of this cancer, compared to those bearing wild type (WT) variants of these SNPs (OR = 2.1, 95% CI = 1.2–3.6, *p* = 0.005) [39]. However, both associations (rs2059520 or rs2303428) were confined to heterozygous genotypes; neither of the homozygous variant alleles was associated with endometrial cancer and this fact precludes clear interpretation. Rs2303428 is the most studied variant in *MSH2* gene, being associated with various types of cancer (CRC, melanoma, lung, non-Hodgkin lymphoma, glioblastoma and BC; for details see the Supplementary Materials). 

**Figure 5:**
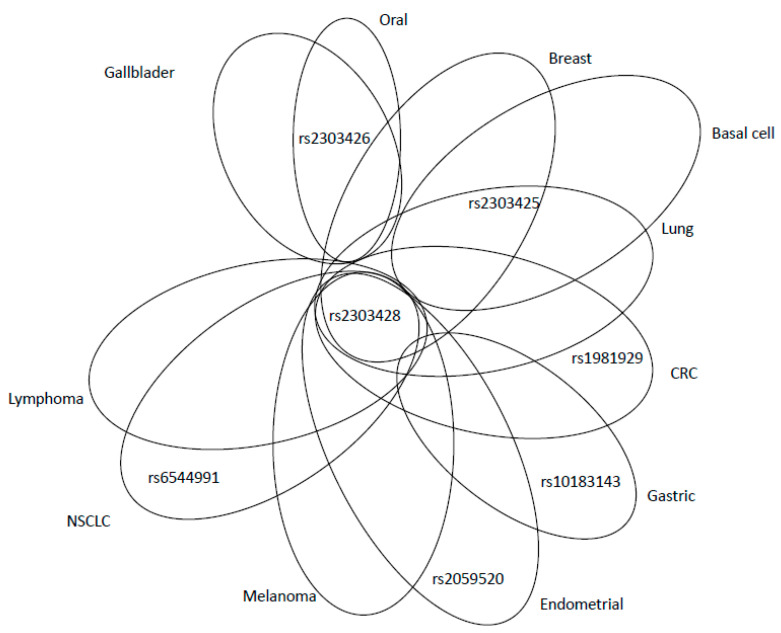
Venn diagram showing relevant MSH2 SNPs associated with adverse effects, in overlap among different cancer types or peculiar for specific malignancies. CRC: colorectal cancer; NSCLC: non-small-cell lung cancer.

**Figure 6:**
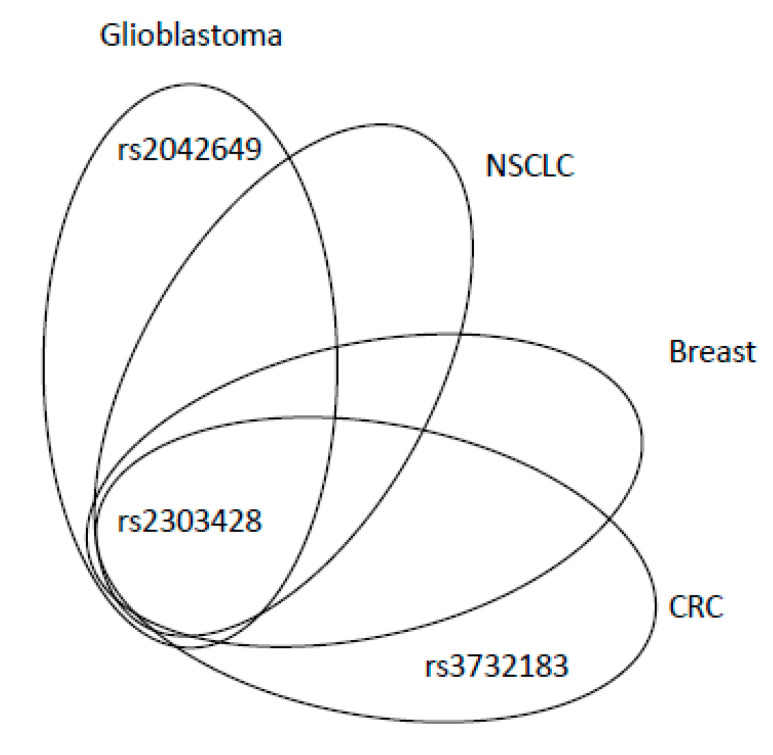
SNPs of *MSH2* with protective effects for the development of various tumours. CRC: colorectal cancer; NSCLC: non-small-cell lung cancer.

There are no exonic SNPs in *MLH2* that are associated with the risk of solid cancers. However, SNPs localised in the intronic sequences may significantly impact the development of cancer, OS and the efficacy of the treatment. The studies on the *MLH2* gene demonstrated how the genetic variants in the non-exonic sequences of the genome might play an important role in human malignancies. An overview of adverse and protective effects of SNPs is shown in Figure 5 and Figure 6, respectively. The most intensively studied rs2303428 was found to be associated with seven different types of cancer. The other SNPs were considered as risk factors in fewer cancer types (Figure 5). Again, rs2303428 had protective effects in more types of cancer, suggesting that one SNP may have both an adverse (Figure 5) and protective role in cancer (Figure 6). The reason of this ambiguity is still unknown, possible explanations are complex gene–gene and gene–environment interactions.

### 3.4. MSH3 Gene (MutS Homolog 3)

The *MSH3* gene is located on chromosome 5 at the q14.1 site. There are three known transcripts of *MSH3*, the longest one, consisting of 24 exons, encodes a 1137 amino acid long protein. The dsSNP database summarises 53,628 SNPs in humans, 46 of them were cited in PubMed. MSH3 forms a heterodimer with MSH2 to generate a MutSβ protein complex. MutSβ is involved in post-replicative DNA MMR. It initiates binding to mismatches and then forms an interaction with a MutLα protein complex. In general, MutSβ preferentially recognises large insertion and deletion loops, up to 13 nucleotides long. The somatic mutations can be found in approximately 50% of MMR-deficient CRCs [89]. MSH3 also interacts with the proliferating cell nuclear antigen (PCNA), which is important for the localisation of MutSβ into replication foci. PCNA assists in the initiation step of MMR, guiding the MutSβ complex and other components of the MMR system to free termini in newly replicated DNA [90]. Interestingly, overexpression of *MSH3*, which can also occur in the tumour, causes substantial changes in the levels of MutSα and MutSβ complexes. MutSα is depleted and MSH6 is degraded. This can be explained by high levels of MSH3, which sequester MSH2, important for the formation of MutSα complex [91]. 

A nonsynonymous SNP, rs184967 (c.2846A>G; Gln940Arg), was found to be associated with the risk of proximal colon cancer (*p* = 0.005). Individuals bearing this SNP along with a higher intake of processed meat did not exert higher risk of CRC than individuals with WT variant. Patients with either the heterozygous G/A (OR = 1.10, 95% CI = 0.82–1.47) or homozygous A/A genotypes (OR = 1.26, 95% CI = 0.96–1.64) had worse progression-free survival (PFS) than those with the G/G genotype (OR = 1.0; 95% CI = 15.03–37.71, *p* = 0.04) [92]. One of the most studied SNPs in *MSH3* is a frame-shift-coding SNP, rs26279 (c.3133G > A; Ala1045Thr). Genotype frequencies of this variant were found to be higher in CRC patients (*p* = 0.04) [93]. In a Portuguese population, gene–gene interaction between *MSH3* Ala1045Thr and *MSH6* Gly39Glu conferred the decreased BC risk (*p* = 0.01) [73]. Several studies, by analysing rs26279, observed increased risk of CRC, squamous cell cancer (SCC), non-small cell lung cancer (NSCLC), BC, hepatocellular carcinoma and oesophageal cancer (see Supplementary Materials). In the meta-analysis, the authors assessed 11 publications comprising 3282 cases and 6476 controls and confirmed that this SNP increases the risk of various types of cancer, mainly CRC and BC, especially in Europeans and Asians [94] (see Supplementary Materials). An intronic SNP rs863221 (c.1763+1841T>G) was studied as prognostic marker on the cohort of 2060 CRC patients from the European population using a SNP tagging approach. Among 68 SNPs in seven MMR genes, 10 appeared significant in the univariate analysis (*p* < 0.05), but after adjustment for sex, age, stage and diagnosis, only rs863221 in *MSH3* gene remained associated with better OS in CRC patients (HR = 0.59, 95% CI = 0.42–0.82, *p* = 0.001) [95]. Two additional SNPs (rs836808 and rs1105524) were associated with an increased risk of BC and NSCLC. The first study discovered that an intronic SNP rs836808 (c.1174-176G>A) was associated with a higher risk of BC patients (1.06–1.19, *p* = 0.008). This multicentric consortium project collected data from 28 publications, including 70,917 SNPs, 46,450 patients and 42,461 controls of European origin [96]. The second SNP (rs1105524, c.-35A>G) was studied on Chinese NSCLC patients, treated with platinum-based chemotherapy. Patients with either the heterozygous G/A or homozygous A/A genotypes had worse PFS (median survival time 14.3 months, 95% CI = 9.80–18.75) than patients with G/G genotype, (median survival time 26.4 months, 95% CI = 15.03–37.71, *p* = 0.04) [97].

**Figure 7:**
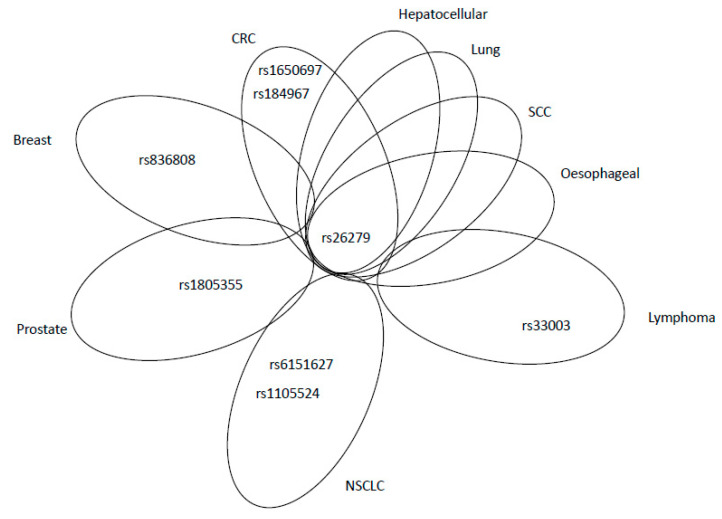
Venn diagram showing relevant MSH3 SNPs associated with adverse effects, in overlap among different cancer types or peculiar for specific malignancies. CRC: colorectal cancer; SCC: squamous cell carcinoma; NSCLC: non-small-cell lung cancer.

A significant association between rs33003, an intronic SNP (c.3303-436A>G), and diffuse large B cell lymphoma was found in a case–control study of subjects of European ancestry (*p* = 0.01) [98]. This study encompassed 118 genes, 599 SNPs and 1116 samples. However, the authors discussed that this association was not replicated by another North American study [99], indicating this result as false positive. The overview of adverse effects of *MSH3* SNPs is presented in Figure 7; the most studied rs26279 was associated with worse outcome of various cancer types. However, rs26279 had a protective effect on the development of BC. It seems that the evolution of BC differs in some molecular mechanisms from other types of cancer, with the significant protective role of SNPs in MMR genes. Rs836808 had an opposite, adverse effect on the BC development.

### 3.5. MSH4 Gene (MutS Homolog 4)

The *MSH4* gene is located on chromosome 1 at the p31.1 site. There is only one transcript of the gene identified, consisting of 20 exons and encoding a 936 amino acid long protein. The dsSNP database covers 28,103 SNPs in humans, but only 10 of them were cited in PubMed. The physiological functions of MSH4 protein are similar to those of MSH5: MSH4 is important for meiotic recombination, specifically required for reciprocal recombination and segregation of homologous chromosomes during meiosis I [100]. In the yeast *Saccharomyces cerevisiae*, Msh4 and Msh5 act specifically to facilitate crossovers between homologous chromosomes [101]. The Msh4/Msh5 complex binds and stabilises double Holliday junctions and promotes their resolution into crossover products. The ortholog of *MSH4* in *Caenorhabditis elegans*, *him-14*, is important for crossing over in meiosis and loss of *him-14* results in lack of chiasmata between homologs and consequent mis-segregation [102]. MSH4 forms a unique and exclusive heterodimer with MSH5, but also with MLH1 and MLH3 [74,103,104]. It appears that MSH4 acts during meiosis to direct the recombinational repair of DNA double-strand breaks produced in prophase I of meiosis I [105], and MSH4–MSH5 dimers are important for mitotic and meiotic DNA double-strand break (DSB) repair [106,107]. MSH4 participates in the maintenance of genomic stability by restricting the use of error-prone non-homologous end joining following DSBs [108].

Among all SNPs cited in PubMed, only rs5745325 (c.289G>A; Ala97Thr) was associated with the increased risk of BC [73]. Interestingly, this SNP alone has no impact on the BC risk, but the authors described a gene–gene interaction between *MSH4* Ala97Thr and *MLH3* Leu844Pro (rs5745325 and rs175080, respectively), resulting in an increased BC susceptibility (A/G genotype of *MSH4* with A/A genotype of *MLH3* (OR = 2.35, 95% CI = 1.23–4.49, *p* = 0.01), G/G genotype of *MSH4* with A/A genotype of *MLH3* (OR = 2.11, 95% CI = 1.12–3.98, *p* = 0.02) and G/G genotype of *MSH4* with A/G genotype of *MLH3* (adjusted OR = 1.88, 95% CI = 1.12–3.15, *p* = 0.02)). The study was conducted in a Caucasian Portuguese population. 

### 3.6. MSH5 Gene (MutS Homolog 5)

The *MSH5* gene is located on chromosome 6 at p21.33 site. There are 19 transcripts of the gene; the longest one contains 25 exons, encoding an 834 amino acid long protein. The dsSNP database comprises 6385 SNPs in humans, 22 of them were cited in PubMed. All findings in mice and humans indicate that MSH5 plays an important role in the meiotic recombination. Mice with a null *MSH5* mutation (*MSH5*^−/−^) are viable but sterile. Phenotypically they display a disruption of chromosome pairing leading to defects in prophase I of meiosis [109]. The experiments in *Caenorhabditis elegans* showed that mutations in *MSH5* did not affect the repair capacity of the MMR system, but they caused defective crossovers between homologous chromosomes [110]. However, recent studies showed that MSH5 is also important for the stability of mitochondrial genome after oxidative stress and for the repair of double-strand break (DSB) after chemotherapeutic treatment [111,112]. As MSH5 interacts with other MMR proteins, it is a part of the MMR system [103].

There are only a few SNPs in *MSH5* with significant implications in the neoplasia. The G allele of rs707938 (c.2148A>G; Gln716Gln) was proved in one study to be associated with the increased risk of lung cancer [113]. The authors also described patients with either homozygous variant A/A or heterozygous A/G variant of rs3131379 (c.813-45G>A) as susceptible towards lung cancer, this tendency was more pronounced among individuals who carried UNG (uracil DNA glycosylase) rs246079 SNP (c.802-574A>G). The other four original studies confirmed that rs3131379 is strongly associated with lung cancer (see Supplementary Materials). The above finding was summarised in the meta-analysis by Timofeeva et al., which included 14,900 cases and 29,485 controls of European descent, based on 16 previously reported GWAS studies undertaken by nine analytical centres [114]. Yi et al. found by the yeast two-hybrid assay that the presence of SNP rs2075789 (c.85C>T; Pro29Ser) significantly weakens the interaction of MSH4-MSH5 complex [115]. The last two studies, which were carried on Chinese populations, revealed that rs707938 increased the risk of hepatocellular carcinoma; rs707939 (c.1326+36C>A) had the negative effect in lung cancer patients, increasing the overall toxicity to platinum-based chemotherapy [76,116]. Recently, the authors investigated the role of DNA repair genes in LC patients by a multilevel association study with 1655 single nucleotide polymorphisms (SNPs) in 211 DNA repair genes on 6911 individuals pooled from four genome-wide case–control studies. Single SNP association corroborates the association described in previous reports for *MLH5* rs3131379 (*p* = 3.57 × 10^−5^) [117]. Additional evidence on the concerted function of *MSH5* gene variant with a decreased CpG methylation was reported for LC patients [118].

A GWAS was conducted in 1952 LC cases and 1438 controls. By pooling data with two other GWAS (5095 cases; 5200 controls) and with replication in an additional 2484 cases and 3036 controls the authors identified rs3117582, BAT3-*MSH5*; Pcombined = 4.97 × 10^−10^ [119]. In a cross-cancer analysis, the authors analysed 60,297 single nucleotide polymorphisms at 229 DNA repair gene regions, using data from the NCI Genetic Associations and Mechanisms in Oncology (GAME-ON) Network. Included were the data from 32 GWAS on 48,734 controls and 51,537 cases across five cancer sites (BC, colon, lung, ovary and prostate). The authors identified three susceptibility DNA repair genes: *RAD51B* (*p* < 5.09 × 10^−6^); *MSH5*, rs3115672 (*p* < 5.09 × 10^−6^); and *BRCA2* (*p* = 5.70 × 10^−6^) [120]. SNPs in *MSH5* are associated with fewer types of cancer, probably due to lack of relevant studies on this gene. 

### 3.7. MSH6 Gene (MutS Homolog 6)

The *MSH6* gene is located on chromosome 2 at p16.3 site. There are 15 transcripts of the gene; ten of them are protein-coding. The longest transcript has 12 exons, encoding a 1360 amino acid long protein. The dbSNP database covers 11,472 SNPs in humans, 48 of them were cited in PubMed. MSH6 heterodimerises with MSH2 protein to form MutSα complex. Its pivotal role is to recognise base–base mismatches, as well as dinucleotide insertion–deletion loops. When the mismatched region in the DNA is found, it exchanges ADP by ATP to repair DNA mismatch together with the MutLα complex [121]. Mutations in the *MSH6* gene are frequently associated with HNPCC, CRC and endometrial carcinoma [122].

The most frequently studied SNP of *MSH6* is rs1042821 (c.116G>A; Gly39Ala), which was found to have controversial effects in cancer patients. While together with *MSH3* rs26279 (Ala1045Thr) it has a protective role in BC patients, alone it acted as a risk factor for BC [73,123]. SNP rs1042821 was associated with decreased risk of primary hepatocellular cancer, and together with *MSH3* rs26279 (Ala1045Thr) with decreased risk of oesophageal cancer; patients carrying the heterozygous G/A genotype of rs1042821 did not display higher risk of CRC (OR = 1.65, 95% CI = 1.01–2.69, *p* = 0.44) [124,125]. A recent case–control study comprising 106 thyroid cancer patients and 212 age- and gender-matched controls revealed that *MSH6* rs1042821 variant homozygotes exhibited higher risk of this cancer (OR = 3.42, CI = 1.04–11.24, *p* = 0.04). Despite the fact that this association was especially evident for the follicular histotype and female sex, the outcomes need to be replicated [126]. Homozygous carriers had a higher risk of CpG island methylated phenotype (CIMP+) in colon cancer (OR = 2.20, 95% CI = 1.10–4.20) than those with WT genotype [76,124,127,128]. A very significant association with the pancreatic cancer was also found (*p* ≤ 0.002) for A/G or A/A genotype [129]. A protective effect of exonic region rs1800932 (c.276A>G, Pro92Pro) in CRC onset was observed in the European population comprising 1785 cases and 1722 control subjects [64]. The intronic SNP rs3136228 (c.-152-405T>G) was identified as a negative marker associated with pronounced adverse effects of chemotherapy. It aggravated the neutropenia in CRC patients treated with FOLFOX4 regimen, decreased the responsiveness to fluoropyrimidine-based chemoradiotherapy in patients with rectal cancer and decreased the OS of prostate cancer patients [68,130,131]. The most significant SNP with adverse effect on cancer risk was rs1042821, associated with pancreatic, prostate and CRC, whereas it had protective roles in the BC, hepatocellular and oesophageal cancers.

### 3.8. PMS1 Gene (Postmeiotic Segregation Increased 1)

The *PMS1* gene is located on chromosome 2 at 2q32.2 locus. The largest mRNA transcript encodes a 932 amino acid long protein, PMS1, which interacts with MLH1 to form a MutLβ protein complex. The *PMS1* gene has 22 splicing and 19 protein-coding variants. The longest transcript consists of 12 exons, coding for a protein with 756 amino acids. Up until now, 21,872 SNPs were identified, but only 32 of them were cited in PubMed database. In fact, only a few SNPs in *PMS1* are associated with carcinogenesis, and are mainly seen in patients with the immunological pathologies. The role of different SNPs in this gene in HNPCC syndrome is still disputed and SNPs are classified as possibly damaging. 

Intronic SNP rs5742933 (c.-24G>C) was described as a silencer of exon splicing predicted in a bioinformatic study by PupaSuite software. Based on the PolyPhen scores and availability of three-dimensional structures, structure analysis was carried out with the major mutations that occurred in the native protein [132]. Dong et al. examined 218 SNPs in 50 DNA repair genes in 568 NSCLC patients. Among all those genes, SNP rs5742933 in *PMS1* was associated with a worse prognosis (HR = 1.89, 95% CI = 1.17–3.06, *p* < 0.0001) [133]. Another intronic SNP, rs5742938 (c.-21+639G>A), was associated with increased risk of oesophageal cancer in South Africans with mixed ancestry (G/G versus A/A or A/G: OR = 1.73, 95% CI = 1.07–2.79, *p* = 0.027), but with no effect on the *PMS1* mRNA expression levels [127]. This variant has never been associated with any cancer except for this study. As it was discussed for the *MSH2* gene, no exonic SNPs in *PMS1* were found to be pathogenic and associated with the increased risk of any cancer. It is disputable why some regions of the human genome are more prone to variations, and it is likely the nucleotide sequence and position on the chromosome play an important role. It is surprising mainly for the *MSH2* gene, which is by far the longest MMR gene. Only two SNPs were found to have adverse effects in NSCLC and oesophageal cancer development. No protective SNP has been described yet.

### 3.9. PMS2 (Postmeiotic Segregation Increased 1, Homolog 2) Gene

The *PMS2* gene is located on chromosome 7 at the p22.1 locus. The largest mRNA transcript encodes an 862 amino acid long protein PMS2, which interacts with the MLH1 to form a MutLα protein complex. Assembly of the MutL–MutS–heteroduplex ternary complex in the presence of Replication factor C (RFC) and PCNA is sufficient to activate the endonuclease activity of PMS2. PMS2 interaction with DNA polymerase III is essential for its recruitment to the site of MMR. The *PMS2* gene has seven splicing variants and the largest one has 15 exons. Up until now, 11,866 SNPs were observed, 39 of them cited in the PubMed database. SNPs in the *PMS2* gene are mainly associated with HNPCC syndrome. For example, seven HNPCC patients were studied for SNP rs1805321 (c.1408C>T, Pro470Ser) in exon 11. Loss of heterozygosity (LOH) was detected in a tumour with decreased signal from the common C allele [134]. Individuals with ovarian cancer and bearing allele A of intronic SNP rs2228006 (c.1621G>A) displayed better OS (HR = 0.84, 95% CI = 0.71–0.99, *p* = 0.04) [135]. However, the authors concluded the study as probably false positive after adjusting for multiple comparisons. Among the nine SNPs investigated in *PMS2* by Li et al., rs2228006 was associated with pancreatic cancer risk (*p* = 0.036) [78]. The intronic SNP rs7797466 (c.24-1121C>T) was associated with a 1.17-fold increase in ovarian cancer risk (95% CI = 1.03–1.33, *p* = 0.013), and haplotype analysis showed significant differences in frequencies between cases and controls (*p* = 0.005) [136]. Interestingly, common G/G variant of rs7797466 increased the risk of CRC when combined with G/A variant of *MSH6* SNP rs1042821, as investigated in a Polish case–control study of 200 patients and 200 controls (OR = 1.65, 95% CI = 1.01–2.69, *p* = 0.44) [124]. The SNP rs7797466 was also significantly associated with pancreatic cancer (OR = 1.44, 95% CI = 1.14–1.81, *p* ≤ 0.002) on the cohort of 706 patients [129]. Rs7797466 was the most studied SNP of *PMS2* and found to be associated with increasing risk of pancreatic, CRC and ovarian cancer. No protective roles of *PMS2* SNPs have been described yet.

### 3.10. PMS2P1 (Postmeiotic Segregation Increased 1 Homolog 2, Pseudogene 1) Gene

The *PMS2P1* gene, also known as *PMS2L1*, is located on chromosome 7 at the q22.1 site. It is a pseudogene, corresponding to the first five exons of *PMS2*, encoding a 440 amino acid protein, PMS2P1. According to the dbSNP database, 1445 SNPs were found in human *PMS2P1*, but none of them were cited in the PubMed database or analysed so far. *PMS2P1* was characterised by two independent groups, and no protein interaction with human MLH1 was observed [137,138,139]. Only one study described the association of a nonsense homozygous mutation in the *PMS2P1* gene (c.2428C>T, Arg802X) with a supratentorial primitive neuroectodermal tumour in three siblings, suggesting the involvement of *PMS2P1* mutations in childhood cancer [140]. The authors concluded that although *PMS2P1* mutations in the CRC may be rare, their involvement in childhood cancers is underestimated. Moreover, previously described polymorphisms in *PMS2P1* were, in fact, pseudogene sequence variants of *PMS2*.

### 3.11. PMS2P2 (Postmeiotic Segregation Increased 1 Homolog 2, Pseudogene 2) Gene

The *PMS2P2* gene, also *PMS2L2*, is located on chromosome 7 at q11.23 site. It encodes a 297 amino acid protein, PMS2P2, which shares sequence homology with the 5′region of PMS2. *PMS2P2* was studied by yeast two-hybrid assay and no interaction with the human MLH1 protein was identified [139]. The authors suggested that PMS2P2 may play a significant role in MMR due to sequence similarity with PMS2. According to the dsSNP database, 1299 SNPs were found in human *PMS2P2*, but none of them was cited in PubMed database or analysed. 

## 4. Role of MMR Variants in Sporadic Cancer 

Despite the gaps in the understanding of the functional consequences of SNPs, it has been observed that most of the pathogenic SNPs in MMR genes affect regulatory gene sequences. Mitchell et al. suggested that changes in the regulation of gene expression may have a more significant effect on carcinogenesis than structural changes of the protein product [141]. We may consider at least two molecular mechanisms affecting the regulation of gene expression: changes in gene promoter activity and synthesis of functionally different splicing variants. 

One mechanism involved in the regulation of gene expression is represented by promoter methylation. In the majority of sporadic CRC with MSI-high phenotype, the MMR system is inactivated mainly by hypermethylation of *MLH1* promoter [142]. In this context, the possible interference of rs1800734 polymorphisms, residing within the core promoter region of *MLH1*, with methylation should not be underestimated. The arising errors may alter the length of repetitive DNA sequences (microsatellites) and result in erroneous transcription of tumour suppressor genes and growth factor receptors [5,6]. This molecular mechanism was also observed in many other malignancies: endometrial and ovarian, laryngeal squamous cell and squamous cell cancer of the lung [143,144,145,146]. Different molecular mechanisms, such as allelic imbalance and somatic mutations, probably play a minor role in the pathogenesis of CRC. Geisler et al. observed that absent *MLH1* expression was associated with the loss of expression of the other five MMR genes (*MSH2*, *MSH3*, *MSH6*, *PMS1* and *PMS2*) [144]. Interestingly, a recent study showed that patients with HNPCC exhibit a similar cancer prognosis as those with sporadic CRC with confirmed *MLH1* promoter hypermethylation [147]. *MSH3* promoter hypermethylation was also observed in bladder cancer [47]. The role of SNPs in the regulation of the expression is not clear yet. For example, SNP rs1800734 in the *MLH1* gene was found to affect DNA repair capacity in lung cancer patients via transcription regulation [47]. A significant association was shown between SNP rs1650697 in the *MSH3* gene and sporadic CRC with MSI in the Japanese population [148]. Two polymorphisms, rs3136229 (c.-448G>A) and rs41540312 (c.-159C>T), in the *MSH6* promoter region affected the interaction of Sp1 transcription factor with the *MSH6* promoter and increased the sensitivity of the promoter to DNA methylation silencing in vitro [149]. The authors suggested that these SNPs could be preferential sites of de novo methylation but may also facilitate the binding of other factors to newly methylated DNA, which then amplifies the silencing of *MSH6* expression.

The second molecular mechanism affecting the proper function of MMR is a synthesis of different splicing variants due to intronic SNPs. For example, SNP rs2303428 (c.2006-6T>A) in *MSH2* intron 12 was studied in meta-analysis for its role in carcinogenesis [150]. The SNP was found to be only associated with the increased risk of non-Hodgkin’s lymphoma (OR = 1.62, 95% CI = 1.06–2.47). However, the authors concluded that this SNP causes a different effect on different types of cancer and further investigation is needed due to the lack of data from the reviewed studies and missing studies from African populations. One of the first functional studies demonstrated that three SNPs in *PMS2* (rs2228007, rs1805318 and rs1805324) cause defective protein–protein interaction of PMS2 with MLH1. These SNPs encode the interaction domain for MLH1 [151]. However, the frequencies of these SNPs are below 5% in the European population.

MMR genes are widely expressed in different types of cells, but organ-specific or tissue-specific mechanisms may occur and become relevant only for some types of cancers. Further investigations are warranted to understand the additional mechanisms by which SNPs in MMR contribute to carcinogenesis in solid tissues. The overview of SNPs with known phenotype is shown in Table 2.

## 5. SNPs in MMR Genes and Therapy

MMR was indicated to participate in response to many drugs used in anticancer treatment. For example, cytotoxicity of methylating agents (due to O^6^-methylguanine) is mediated primarily by MMR system components, which recognise mismatches generated by these agents and activate cell cycle arrest and apoptosis [152]. AGEO, a recent large multicentric study, investigated the efficacy of fluoropyrimidine with and without oxaliplatin in a large cohort of MMR-deficient colon cancer patients, as MMR-deficient colon cancer is resistant to 5-fluorouracil adjuvant chemotherapy while preliminary data suggest chemosensitivity to oxaliplatin [153]. Therefore, SNPs in MMR genes may alter the molecular mechanisms of drug response and thus change the responsiveness of patients to treatment. Analysis of SNPs is now considered as an approach helping the oncologist to set a more specific, personalised therapeutic regimen for every single patient and yielding important information for the development of new drugs. Russo et al. supposed that the different responses to a particular drug are not only due to clinicopathological or environmental factors, but also due to the ethnic origins and particular genotype of a patient [154]. Other studies proved that SNPs in MMR genes may substantially change efficacy, toxic effect and the outcome of cancer therapy. For example, SNP rs2303428 in *MSH2* was found to be associated with the adverse response and outcome in O^6^-guanine-based therapy and in radiotherapy. The presence of the C allele was significantly overrepresented in acute myeloid leukaemia patients previously treated with O^6^-guanine alkylating agents, compared with controls (OR = 4.02, 95% CI = 1.40–11.37) [155,156]. The expression of MMR genes may be significantly altered after chemotherapy, as reported by Murata et al. [153]. The authors observed a strong correlation between pre-surgical chemotherapy and reduced *MLH1* expression. They suggested that the silencing may arise due to promoter hypermethylation induced by chemotherapy. Thus, patients carrying SNPs that increase promoter hypermethylation can differently respond to the therapeutic regimen. The reduced expression of *MLH1* conferred an advantage for the progression of tumours and hypermethylation of the promoter was linked with clinical stage and lymphatic metastases. Park et al. studied the associations between SNPs and chemotherapy-based treatment of CRC patients [157]. The authors found that SNP rs1625649 in the O^6^-Methylguanine-DNA methyltransferase (*MGMT*) gene, involved in direct reversal repair, significantly impaired OS of CRC patients treated with oxaliplatin-based therapy, but the combined A/G + G/G genotype of the *MSH2* rs3732183 (c.1661+12G>A) was associated with better OS (*p* = 0.02) [157]. Other studies on Korean populations showed the protective effect of this SNP in CRC and lung cancer. The presence of the G/G genotype of rs3732183 was associated with significantly decreased risk of lung adenocarcinoma compared to A/A carriers (*n* = 432 patients, *n* = 432 controls, OR = 0.59, 95% CI = 0.40–0.88, *p* = 0.01). The CRC patients with the combined G/G + A/G genotype revealed better OS than patients with A/A genotype (*n* = 379 cancer patients, HR = 0.50, 95% CI = 0.26–0.98, *p* = 0.042) [158,159]. Another SNP, rs6544991 (c.*251+2441A>C), in the *MSH2* gene was associated with the increased toxicity of platinum-based chemotherapy within NSCLC treatment [116]. This study demonstrated that the rs6544991 C/C genotype was associated with gastrointestinal toxicity; *MSH3* rs6151627 (c.580-380A>G), rs6151670 (c.1340+8303C>G) and rs7709909 (c.1341-20102C>T) were associated with hematologic toxicity; and *MSH5* rs707939 (c.1326+36C>A) was associated with gastrointestinal and overall toxicity. The other study focused on the role of SNPs in MMR genes (*MLH1*, *MSH2* and *MGMT*) in melanoma patients after temozolomide and dacarbazine treatment, including comparison with five melanoma cell lines [160]. The authors observed the decreased expression of the *MLH1* gene in patients bearing SNP rs1799977 and found five new SNPs in *MLH1*. The presence of SNPs and promoter methylation of MMR genes was identified as a major cause of melanoma treatment resistance. The role of rs1799977 in the *MLH1* gene was also studied in B cell lymphoma patients treated with R-CHOP21 (rituximab, cyclophosphamide, doxorubicin, vincristine and prednisolone administered every 21 days) and platinum-based therapy. Patients carrying either the A/G or G/G genotype showed an increased risk of death (HR = 3.21, *p* < 0.001) compared to those carrying the A/A genotype [63]. This study vindicated that MMR components can significantly regulate the genotoxicity of doxorubicin and platinum-based therapy. The role of variants in MMR genes in CRC development was studied in the Czech population, comprising 1095 cases and 1469 controls [15]. Carriers of the C/C genotype of rs108621 (c.*3148A>G) in the 3’UTR of *MLH3* (a microRNA binding site), after 5-fluorouracil-based (5-FU) chemotherapy, showed a significantly increased OS compared to those with either C/T or T/T genotype (*p* = 0.05). Moreover, heterozygous carriers had a higher risk of relapse (*p* = 0.03). Patients carrying the C/C genotype for *MSH6* rs1800935 (Asp180Asp) and not undergoing 5-FU-based chemotherapy showed a decreased risk of recurrences (*p* = 0.03). In another study, the same authors investigated the epigenetic mechanisms and the expression of MMR genes comparing the tumours and adjacent mucosa tissues in sporadic CRC patients. They observed different expression according to tumour localisation—colon tumours had higher expression of MMR genes in comparison to rectal cancer (*p* = 0.02). Only 9% of tumours were positive for the methylation of the *MLH1* gene [161]. The SNP rs3136228 in *MSH6* was studied for its contribution to CRC development. A prospective study in CRC patients treated with FOLFOX4 regimen revealed possible association of rs3136228 in *MSH6* with grade 3 neutropenia toxicity (*p* = 0.07). This SNP may affect the MMR activity in non-malignant cells and therefore modulate the genotoxic effect of FOLFOX therapy [131]. The same group showed that this SNP is in the upstream region of the gene and seems to affect the binding with the transcription factor Sp1, followed by decreased expression of *MSH6* and thus MMR efficiency [68]. The strongest association (*p* < 0.01) between genetic variants and pathological response to neoadjuvant treatment of rectal cancer was observed particularly for rs3136228 in *MSH6* gene in the subgroup of patients undergoing standard treatment-5-FU combined with a dose of 5040 cGy of radiotherapy. The other SNPs in 21 genes did not show any significant association. The overview of SNPs evaluated in clinical studies is presented in Table 3.

## 6. Conclusions

The overall conclusion is that the role of SNPs in the MMR pathway in carcinogenesis is not clear yet. Available publications reveal substantial variability among SNPs in various types of cancer. Some genetic variants have a beneficial effect in a particular cancer and some of them are pathological or often described as possibly damaging. The pool of available studies contains reports of various size and quality and the studies based on a limited number of patients may bias the overall outcome. The manuscripts considered in our review disclose for the first time the implication of same variants in MMR genes (such as in *MLH1*, *MSH2*, *MSH3* and *MSH6*) in the risk of different cancers. These SNPs will deserve attention in the future studies.

One of the main problems is a lack of functional studies which may provide evidence of the function and ultimate pathogenicity of a particular SNP. Apparently, most of the pathogenic SNPs in the group of MMR genes affect regulatory sequences, important for proper gene expression. Therefore, it seems that these changes are more or equally involved in carcinogenesis than structural changes in the relevant protein product. An additional aspect worthy of consideration is tumour heterogeneity. According to some GWAS studies, SNPs correlate with the tumour stage or grade. In general, the authors of GWAS studies did not focus on the possible mechanism by which the particular SNP modulated the function of MMR gene. 

Results from all available studies suggest that single SNPs in MMR genes may contribute to sporadic cancer susceptibility, cancer progression and may affect the response to therapy. In our recent review, we noted that individual genetic variation in biotransformation, DNA repair and mitosis regulating genes exerted small modulating effects on chromosomal aberrations; in interactions their effect was more pronounced [162]. The same may apply to the role of MMR gene variants in complex diseases, such as cancer. The interactions (mainly binary) between MMR SNPs are only fragmentarily addressed on relatively small studies. Further research in this direction may provide important outcomes.

Extensive interactions between proteins of distinct DNA repair pathways need to be considered in evaluating the DDR-related risk as well [30], understanding these interplays may serve as prognostic and predictive factors for several malignancies.

## Figures and Tables

**Table 1:** The DNA error frequencies during the replication. The mismatch repair system (MMR) appears important in ensuring the DNA replication fidelity [20].

Mechanism	Error Frequency
Base pairing	10^−1^ to 10^−2^
DNA polymerases (base selection, proofreading)	10^−4^ to 10^−5^
Accessory proteins	10^−7^
Mismatch repair	10^−10^

**Table 2:** The overview of SNPs in EUR population (F ≥ 5%) with known phenotype in the tumour development: B: benign, D: deleterious, A: ambiguous, P: pathogenic.

Gene	SNP ID	AA Change	WT-MT	Ancestral Alelle	Gene Region	Gene Position	Phenotype	F in EUR Pop.
*MLH1*	rs1540354	-	A-T	T	intron	c.307-1403A>T	B	16%
	rs1558528	-	C-A	A	intron	c.67+955C>A	P	56%
	rs2286939	-	T-C	C	intron	c.1038+86T>C	D	56%
	rs4647250	-	T-C	T	intron	c.453+544T>C	D	44%
	rs1799977	Ile219Val	A-G	A	Exon 8	c.655A>G	A	33%
	rs1800734	-	A-G	G	5′ prime	c.-93G>A	A	27%
	rs2286940	-	C-T	C	intron	c.1410-169C>T	B	44%
	rs9852810	-	G-A	G	intron	c.1410-1306G>A	P	44%
	rs4647269	-	C-T	C	intron	c.791-1406C>T	A	44%
*MLH3*	rs108621	-	A-G	T	UTR-3	c.*3148A>G	B	45%
	rs175080	Leu844Pro	C-T	G	Exon 2	c.2531C>T	A	46%
	rs28756990	Val741Ile	G-A	G	Exon 2	c.2221G>A	P	5%
	rs175057	-	G-A	T	intron	c.4012-37G>A	P	54%
*MSH2*	rs1981929	-	G-A	A	intron	c.1277-118G>A	P	39%
	rs2042649	-	T-C	T	intron	c.2635-214T>C	P	9%
	rs2059520	-	A-G	A	intron	c.2006-265A>G	P	36%
	rs2303425	-	T-C	T	5′ prime	c.-68-50T>C	P	15%
	rs2303426	-	C-G	G	intron	c.211+9C>G	P	57%
	rs2303428	-	T-A	T	intron	c.2006-6T>A	A	9%
	rs3732183	-	G-A	G	intron	c.1661+12G>A	B	30%
	rs6544991	-	A-C	A	intron	c.251+2441A>C	B	22%
	rs10183143	-	T-C	T	intron	c.1661+90T>C	D	6%
*MSH3*	rs1650697	Ile79Val	A-G	C	Exon 1	c.235A>G	D	24%
	rs26279	Ala1045Thr	G-A	G	Exon 23	c.3133G>A	A	72%
	rs33003	-	A-G	A	intron	c.3303-436A>G	D	71%
	rs184967	Gln949Arg	A-G	G	Exon 21	c.2846A>G	D	14%
	rs836808	-	G-A	C	intron	c.1174-176G>A	P	25%
	rs863221	-	T-G	T	intron	c.1763+1841T>G	B	41%
	rs1805355	Pro231Pro	G-A	G	Exon 4	c.693G>A	P	5%
	rs6151627	-	A-G	A	intron	c.580-380A>G	B	27%
	rs1105524	-	A-G	G	intron	c.-35A>G	P	32%
	rs6151670	-	C-G	C	intron	c.1340+8303C>G	A	27%
	rs7709909	-	C-T	C	intron	c.1341-20102C>T	A	40%
*MSH4*	rs5745325	Ala97Thr	G-A	G	Exon 2	c.289G>A	P	31%
*MSH5*	rs707938	Gln716Gln	A-G	C	Exon 22	c.2148A>G	P	68%
	rs2075789	Pro29Ser	C-T	G	Exon 2	c.85C>T	D	10%
	rs707939	-	C-A	G	intron	c.1326+36C>A	B	37%
	rs3131379	-	G-A	C	intron	c.813-45G>A	P	7%
	rs707939	-	C-A	G	intron	c.1326+36C>A	A	37%
*MSH6*	rs1800937	Tyr214*	C-A	C	Exon 4	c.642C>A	P	10%
	rs1042821	Gly39Ala	G-A	C	Exon 1	c.116G>A	A	18%
	rs1800935	Asp180Asp	T-C	T	Exon 2	c.540T>C	B	29%
	rs3136228	-	T-G	G	intron	c.-152-405T>G	P	65%
	rs1800932	Pro92Pro	A-G	A	Exon 1	c.276A>G	P	18%
*PMS1*	rs5742933	-	G-C	G	intron	c.-24G>C	D	20%
	rs5742938	-	G-A	G	intron	c.-21+639G>A	P	73%
*PMS2*	rs2228006	-	G-A	G	intron	c.1621G>A	P	12%
	rs7797466	-	C-T	G	intron	c.24-1121C>T	P	18%

**Table 3:** The overview of SNPs studied for their role in the therapy of various types of tumours.

Gene	SNP ID	PubMed ID	Reference	Cancer	Cases	Controls	Phenotype
*MLH1*	rs1799977	19203531	[160]	Melanoma	51	0	-
	rs1799977	21156845	[63]	B-cell lymph.	308	0	↓ effect of Dox treatment in folicullar lymphoma
	rs1799977	21156845	[63]	B-cell lymph.	308	0	↓ effect of Pt-based second line treatment
	rs1799977	27608007	[68]	Rectal	280	0	protective factor in rectal c.
	rs1800734	25047469		SCC	185	0	GA genotype ↓ OS in oral squamous cell c.
	rs1800734	25047469		SCC	185	0	AA genotype ↓ OS in oral squamous cell c.
	rs2286940	26743341	[70]	Lymphoid	153	0	sign. predictor of progression-free surv.
*MLH3*	rs108621	24755277	[15]	CRC	1095	1469	CC genotype ↑ survival
*MSH2*	rs2042649	22017238		Glioblastoma	121	0	↑ OS of glioblastoma patients
	rs2303428	22017238		Glioblastoma	121	0	↑ OS of glioblastoma patients
	rs2303428	19741564		Melanoma	51	0	↑ hematologic side effects in melanoma tr.
	rs2303428	20458443		NSCLC	96	0	CC genotype ↑ response to Pt-based therapy
	rs2303428	20708344		Breast	87	0	↑ radiosensitivity in breast c. patients
	rs3732183	20091185	[157]	CRC	94	0	AG/GG genotype ↑ response to therapy
	rs6544991	28093084	[116]	NSCLC	220	0	↑ GIT toxicity of Pt-based chemo
*MSH3*	rs863221	19115210	[95]	CRC	2060	0	↑ OS in patients with CRC
	rs26279	25966119	[97]	NSCLC	180	0	↑ sensitivity to Pt-based therapy in NSCLC
	rs6151627	28093084	[116]	NSCLC	220	0	↑ hematological toxicity in NSCLC patients
	rs1105524	25966119	[97]	NSCLC	180	0	G/A and A/A genotype ↓ OS in NSCLC
*MSH5*	rs707939	28093084	[116]	NSCLC	220	0	↑ hematological toxicity in NSCLC patients
*MSH6*	rs3136228	22868256	[131]	CRC	144	0	grade 3–4 neutropenia
	rs3136228	27608007	[68]	Rectal	280	0	↓ response to F-pyrimidine therapy
	rs3136228	28206966	[130]	Prostate	542	0	↓ OS in prostate c. patients
	rs1800935	24755277	[15]	CRC	1095	1469	CC carriers had ↓ recurrence of CRC

↑ increases the effect, response, survival ↓ decreases the effect, response, survival.

## Supplementary Materials

The following are available online at https://www.mdpi.com/1422-0067/21/15/5561/s1.
